# *Staphylococcus aureus *seroproteomes discriminate ruminant isolates causing mild or severe mastitis

**DOI:** 10.1186/1297-9716-42-35

**Published:** 2011-02-15

**Authors:** Caroline Le Maréchal, Julien Jardin, Gwenaël Jan, Sergine Even, Coralie Pulido, Jean-Michel Guibert, David Hernandez, Patrice François, Jacques Schrenzel, Dieter Demon, Evelyne Meyer, Nadia Berkova, Richard Thiéry, Eric Vautor, Yves Le Loir

**Affiliations:** 1 INRA, UMR1253 Science et Technologie du Lait et de l'Œuf, F-35042 Rennes, France; 2 AGROCAMPUS OUEST, UMR1253 Science et Technologie du Lait et de l'Œuf, F-35042 Rennes, France; 3 ANSES, Laboratoire de Sophia-Antipolis, Unité pathologie des ruminants, F-06902 Sophia-Antipolis, France; 4 Genomic Research Laboratory, Service of Infectious Diseases; University of Geneva Hospitals (HUG), CH-1211 Geneva 14, Switzerland; 5 Ghent University, Faculty of Veterinary Medicine, Merelbeke, Belgium; 6 LVD06, F606902 Sophia-Antipolis, France

## Abstract

*Staphylococcus aureus *is a major cause of mastitis in ruminants. In ewe mastitis, symptoms range from subclinical to gangrenous mastitis. *S. aureus *factors or host-factors contributing to the different outcomes are not completely elucidated. In this study, experimental mastitis was induced on primiparous ewes using two *S. aureus *strains, isolated from gangrenous (strain O11) or subclinical (strain O46) mastitis. Strains induced drastically distinct clinical symptoms when tested in ewe and mice experimental mastitis. Notably, they reproduced mild (O46) or severe (O11) mastitis in ewes. Ewe sera were used to identify staphylococcal immunoreactive proteins commonly or differentially produced during infections of variable severity and to define core and accessory seroproteomes. Such SERological Proteome Analysis (SERPA) allowed the identification of 89 immunoreactive proteins, of which only 52 (58.4%) were previously identified as immunogenic proteins in other staphylococcal infections. Among the 89 proteins identified, 74 appear to constitute the core seroproteome. Among the 15 remaining proteins defining the accessory seroproteome, 12 were specific for strain O11, 3 were specific for O46. Distribution of one protein specific for each mastitis severity was investigated in ten other strains isolated from subclinical or clinical mastitis. We report here for the first time the identification of staphylococcal immunogenic proteins common or specific to *S. aureus *strains responsible for mild or severe mastitis. These findings open avenues in *S. aureus *mastitis studies as some of these proteins, expressed in vivo, are likely to account for the success of *S. aureus *as a pathogen of the ruminant mammary gland.

## Introduction

Mastitis is the first cause of economical loss in milk production worldwide [1] and is a major concern in milk transformation [2]. The problem is however currently hard to tackle for mastitis in dairy cows, sheep and goats. Especially, *S. aureus *mastitis is typically refractory to antibiotic treatment. Prophylactic measures, including the development of an effective vaccine, have so far proven unsuccessful for the control of the disease. *S. aureus *is well-known to produce a large variety of virulence factors (including numerous proteins like toxins or adhesins). Consequently, it induces a large panel of infections, and the clinical acuteness of each infection type may also be variable. For example, *S. aureus *mastitis in dairy sheep ranges from subclinical mastitis to lethal gangrenous mastitis. Such variability relies on staphylococcal virulence factors as well as host factors. Until now, no study has been performed to identify the transcripts and proteins commonly or specifically produced in vivo by *S. aureus *strains during mastitis. To obtain such information using direct transcriptomic or proteomic approaches upon *S. aureus *samples collected within the infection site stumbles on technical bottlenecks such as the low amounts of *S. aureus *cells and the difficulty to localize the infection site within the udder.

Serological proteome analysis (SERPA) is a promising technique that can be used to shed light on the host's immune response to staphylococcal infection. This technique was used to mine new antigen candidates for vaccine development in human infections [3]. SERPA has also been used to identify proteins produced in vivo, during infection [4]. In combination with whole genome shotgun sequencing, SERPA is a powerful tool to identify immunoreactive proteins produced by *S. aureus *during the infection [5].

Genotyping studies indicated that *S. aureus *strains isolated from dairy sheep farms in the south east of France were clonally related and are predominantly represented by a single pulse-field gel electrophoresis (PFGE) type OV/OV' [6,7]. Such close phylogenetic relationship was recently confirmed at the global scale using Multi Locus Sequence Typing and comparative genome hybridization [8,9]. Among strains in this OV/OV' PFGE profile, one was isolated from subclinical mastitis (O46) and another one from gangrenous mastitis (O11). Few genetic differences were identified [10] and their role in the development of mastitis with such different severity has not yet been determined. Moreover identification of the proteins produced by *S. aureus *in mastitis with different severity is an important step to better understand the host-pathogen interactions and to provide targets for the development of efficient prevention or treatment strategies against mastitis. Therefore, we applied SERPA to identify proteins that are produced by both *S. aureus *strains O11 and O46 (core seroproteome) and the ones specifically produced by each strain ("accessory" seroproteome).

## Materials and methods

### Bacterial strains, growth conditions and preparation of protein samples

*S. aureus *strains used in this study are presented in Table 1. *S. aureus *O46 was isolated from a case of ovine subclinical mastitis and O11 from a gangrenous lethal mastitis [10]. Genetic and genotypic background of *S. aureus *O46 and O11 are well-documented. They share the same pulsotype (OV/OV') and are representative of the major lineage found associated to ewe mastitis in south east of France [6,7]. Growth conditions and preparation of protein extracts were as described in Le Maréchal et al. 2009 [11]. Briefly, overnight cultures in BHI were diluted 1:1000 in fresh RPMI 1640 medium (Sigma, Saint Quentin Fallavier, France). RPMI was extemporaneously depleted of iron (and hereafter referred to as iron-depleted RPMI) by adding deferoxamine (0.15 mM) (Sigma). Growth conditions in which there is restriction in the bioavailability of iron can indeed lead to an increase of the expression of virulence factors which are normally expressed in vivo [12]. *S. aureus *strains were grown in 500 mL flasks under agitation (150 rpm) at 37 °C (a flask-to-broth volumetric ratio of 5), for aerobic conditions, or in falcon tubes (50 mL) completely filled with medium and incubated at 37 °C without agitation for anaerobic conditions. Protein samples for supernatant, cell wall or total fraction were prepared exactly as previously described [11].

**Table 1:** *Staphylococcus aureus *strains used in this study

Strain	Type of mastitis	Origin	**Isolated in**:
O11*	gangrenous	south east France	2002
**1628**	gangrenous	south east France	2010
**1624**	clinical	south east France	2003
**1625**	clinical	south east France	2008
**1626**	clinical	south east France	2008
**1536**	clinical	south west France	1998
O46*	subclinical	south east France	2002
**1627**	subclinical	south east France	2008
**O55**	subclinical	south east France	2003
**O117**	subclinical	Corsica. France	2001
**1535**	subclinical	south west France	1998
**O82**	subclinical	south east France	2003

*: O11 and O46 were used for experimental infections and their genome was fully sequenced (see Materials and Methods).

### Genome sequencing of O11 and O46 strains

To facilitate the analysis of SERPA results, the genome of the two strains used in experimental infection was fully sequenced using the Solexa technology (P Mayer, L Farinelli, and E Kawashima, 1997. Patent application WO98/44151) according to the manufacturer's protocol (Illumina, San Diego, CA, USA). Briefly, genomic DNA was physically fragmented by nebulization into 50- to 500-bp fragments. After end repair and ligation of the bar-coded paired-end adaptors, the products were purified on agarose gel to recover products with inserts of ~200 bp. Quality control was performed by cloning an aliquot of the library into a TOPO plasmid and capillary sequencing eight clones per library. The samples were then used to generate DNA colonies using one channel of a paired-end flow cell at dilutions of 4 pM. The flow cell was then submitted to 2 × 74 cycles of sequencing on the genome analyzer. Base calling was performed using the GAPipeline 1.4.0 software; a total of 27.6 million reads (pass filter) were obtained. After bar code selection, 13.9 and 11.8 million reads of 71 bases in length were obtained for the strains O11 and O46 respectively. The pool of sequences obtained was analyzed and assembled using the Edena assembler [13], which resulted in a set of 87 and 96 contigs for O11 and O46, respectively. The gene content of each strain (2787 and 2822 Coding Sequences -CDSs- for O11 and O46, respectively) was thus established and used in protein identification after SERPA. Detailed genome analysis of these strains is described elsewhere (Le Maréchal et al., submitted).

### 2-Dimensional Electrophoresis (2-DE)

Samples (200 μg of proteins for Coomassie blue staining and 50 μg for Western blotting) were precipitated with 2D clean up kit (GE Healthcare, Orsay, France) according to the manufacturer's instructions. Pellets were solubilised in sample solution containing 7 M urea, 2 M thio-urea, 25 mM dithiothreitol (DTT), 4% (w/v) 3-[(3-Cholamidopropyl)dimethylammonio]-1-propane-sulfonate (CHAPS) and 2% (w/v) ampholyte containing buffer (IPG-Buffer 4-7 or 3-10 NL, GE Healthcare). Isoelectric focusing was carried out using pH 4 to 7 (Cell wall and total proteins) or 3 to 10 NL (exoproteins) 13 cm Immobiline Dry Strips on a Multiphor II electrophoresis system (Amersham Biosciences; GE Healthcare, Orsay, France) for a total of 60 kVh using a standard procedure described previously [14]. The second dimensional separation was performed on the Ettan™ DALTtwelve electrophoresis system (GE Healthcare) using 14% acrylamide separating gels without a stacking gel at a voltage of 50 V for 1 h and 180 V for about 7 h. Kaleidoscope Prestained Standards (Biorad) were used as standard. Gels were transferred onto membrane or stained with R250 Coomassie blue (Serva, Heildelberg, Germany) or MS-compatible silver nitrate (Sigma) [15].

### Intramammary challenge with *S. aureus *in ewes

Experimental mastitis was performed according to the Regional Committee for Animal Use and Care (Côte d'Azur, France) and is recorded under reference NCA/2008-14/12-09. Healthy lactating primiparous ewes of Lacaune breeds were selected based on the absence of intramammary infections and milk somatic cell counts below 100 000 cells/mL. Repeated full bacteriological analysis of milk from the two quarters that were going to be infected showed that the ewes were negative for *Staphylococcus *sp. and *Mycoplasma *sp. Absence of nasal carriage for staphylococci was also checked after enrichment and culturing of swab samples of the nares of ewes on selective media, as described previously [6]. At D0, 12 ewes were divided into 2 groups and urethral catheters (Portex^® ^Jackson Cat Catheter, Coveto, France) were inserted into the teat canal after a thorough disinfection of the teat orifice with 70% ethanol. 1 mL PBS containing 20 CFU of *S. aureus *(O11 or O46) was injected through the catheter, which was removed afterwards. Six ewes were thus infected by strain O11 (group O11) and six by strain O46 (group O46). Severity of the mastitis induced in ewes was estimated according to criteria presented in Additional file 1, Table S1. Mastitis was classified as subclinical, clinical, pyogenic and gangrenous. Classification was based on clinical symptoms, presence of *S. aureus *cells and Somatic Cell Count (SCC) in milk.

### Sample processing

Sera from ewes were prepared from blood samples collected aseptically from the jugular vein of the animals at D0, D7, D14, D21 and D28 post inoculation (pi). Briefly, blood samples were kept for 2 h at room temperature before centrifugation. Sera were then stored at -20°C. Milk samples were taken 24 and 36 h pi for bacteriological examination and determination of SCC. Milk was 1/10 diluted and 100 μL of this dilution was plated on selective Rabbit Plasma Fibrinogen Baird-Parker medium to confirm *S. aureus *presence in the mammary gland. SCC was measured with the Fossomatic method [16] to follow the onset of the infection.

### Western blot analysis

Total cell lysates were prepared as previously described [11]. Total protein extracts of *S. aureus *strains O11 and O46 were separated by SDS-PAGE on 12% acrylamide separating slab gels (70 × 100 × 0.5 mm), with a 4% acrylamide stacking gel on a mini-protean III gel system (BioRad, Ivry sur Seine, France) according to Laemmli [17]. Protein migration was performed for 2 h at room temperature at constant 80 V voltage. Samples were diluted in sample buffer and denatured at 100 °C for 3 min. Gels were transferred onto a PVDF membrane (GE Healthcare) at constant 250 mA amperage in Towbin transfer buffer [18] using a Trans-Blot cell (Biorad) for 1.25 h. Membranes were washed three times with Tris Buffered Saline (TBS) at pH 7.5 and saturated in blocking solution (3% non-fat dry milk in TBS with 0.3% Tween 20 (TBS-T)) at 4 °C overnight. After saturation in blocking solution, membranes were washed 3 × 10 min with TBS-T and exposed to the different ewe sera used as primary antibody for 4 h at room temperature. After washing, membranes were incubated with alkaline phosphatase conjugated anti-sheep IgG (Sigma) diluted 1:15,000 in 25 mL blocking solution for 1 h and finally BCIP/NBT (Sigma) was used to visualize immunoreactive proteins, according to the manufacturer's instructions.

### Selection of the hyper-immune sera

Sera samples were analysed by western blotting as described above. Sera sampled on D0, D7, D14, D21 and D28 pi were compared using the mini-protean II Multiscreen apparatus (Biorad) (600 μL of serum diluted 1:10,000 in blocking solution). Immunostained Western blots were scanned using an Image Scanner II (Amersham biosciences) and further analyzed using Image- Quant 1D software. The number, volume and area of bands were taken into account for the analysis. Optimal dilution for the selected sera was determined as described above. Sera and dilutions yielding the best ratio signal/background were selected.

### Identification of immunoreactive proteins

Bacterial proteins separated by 2-DE were transferred onto a PVDF membrane (GE Healthcare) as described above. Series of four gels were migrated and treated in parallel. Three gels were used for immunoblotting, and the fourth one was Coomassie blue-stained for spot matching and further identification. After saturation in blocking solution the membranes were treated with selected sera in blocking solution during 4 h. Then, the membranes were washed with TBS-T and incubated with alkaline phosphatase conjugated anti-sheep IgG (Sigma) diluted 1:15 000 in 25 mL blocking solution for 1 h. Finally BCIP/NBT (Sigma) was used to visualize immunoreactive proteins, according to the manufacturer's instructions. Membranes were scanned using an Image Scanner II (Amersham biosciences) and further analyzed using Image- Master 2D software. Immunoblot profiles for 2-DE-separated proteins were reproducible in at least two individual experiments. Images of the 2D electrophoresis gels and the BCIP-NBT treated membranes were compared to detect immunoreactive proteins. Spots that were absent or had a significantly different intensity in one strain were considered as proteins that differed between O11 and O46. Spots corresponding to proteins of interest were excised and identified using Nano-Liquid Chromatography (Nano-LC) MS/MS analysis.

### Nano-LC MS/MS analysis

Proteins were identified by tandem mass spectrometry (MS/MS) after an in-gel trypsin digestion adapted from Shevchenko [19]. Briefly, gel pieces were excised from the gel, washed with acetonitrile and ammonium bicarbonate solution, and then dried under vacuum in a SpeedVac concentrator (SVC100H-200; Savant, Thermo Fisher Scientific, Waltham, MA, USA). In-gel trypsin digestion was performed overnight at 37 °C and stopped with spectrophotometric-grade trifluoroacetic acid (TFA) (Sigma-Aldrich). The supernatants containing peptides were then vacuum dried in a Speed-Vac concentrator and stored at -20 °C until mass spectrometry analysis. Nano-LC experiments were performed using an on-line liquid chromatography tandem mass spectrometry (MS/MS) setup using a Dionex U3000-RSLC nano-LC system fitted to a QSTAR XL (MDS SCIEX, Ontario, Canada) equipped with a nano-electrospray ion source (ESI) (Proxeon Biosystems A/S, Odense, Denmark). Samples were first concentrated on a PepMap 100 reverse-phase column (C18, 5 μm, 300-μm inner diameter (i.d.) by 5 mm length) (Dionex, Amsterdam, The Netherlands). Peptides were separated on a reverse-phase PepMap column (C18, 3 μm, 75 μm i.d. by 150 mm length) (Dionex) at 35 °C, using solvent A (2% (vol/vol) acetonitrile, 0.08% (vol/vol) formic acid, and 0.01% (vol/vol) TFA in deionized water) and solvent B (95% (vol/vol) acetonitrile, 0.08% (vol/vol) formic acid, and 0.01% (vol/vol) TFA in deionized water). A linear gradient from 10 to 50% of solvent B in 40 min was applied for the elution at a flow rate of 0.3 μL/min. Eluted peptides were directly electrosprayed into the mass spectrometer operated in positive mode. A full continuous MS scan was carried out followed by three data-dependent MS/MS scans. Spectra were collected in the selected mass range 400 to 2 000 *m/z *for MS and 60 to 2 000 *m/z *for MS/MS spectra. The three most intense ions from the MS scan were selected individually for collision-induced dissociation (1+ to 4+ charged ions were considered for the MS/MS analysis). The mass spectrometer was operated in data-dependent mode automatically switching between MS and MS/MS acquisition using Analyst QS 1.1 software. The instrument was calibrated by multipoint calibration using fragment ions that resulted from the collision-induced decomposition of a peptide from β-casein, β-CN (193-209). The proteins present in the samples were identified from MS and MS/MS data by using MASCOT v.2.2 software for search into two concatenated databases: (i) a homemade database containing all the predicted proteins of the *S. aureus *strains O11 and O46 used in this study and (ii) a portion of the UniProtKB database corresponding to the *S. aureus *taxonomic group [20]. Search parameters were set as follows. A trypsin enzyme cleavage was used, the peptide mass tolerance was set to 0.2 Da for both MS and MS/MS spectra, and two variable modifications (oxidation of methionine and deamidation of asparagine and glutamine residues) were selected. For each protein identified in NanoLC-ESI-MS/MS, a minimum of four peptides with MASCOT score corresponding to a *P *value below 0.05 or an Exponentially Modified Protein Abundance Index [21] greater than 0.4 were necessary for validation with a high degree of confidence. For automatic validation of the peptides from MASCOT search results, the 1.19.2 version of the IRMa software was used [22].

### Intramammary infection with *S. aureus *in mice

The animal study was conducted according to current Good Scientific Practice-principles (2000) and approved by the Ethical Committee of the Faculty of Veterinary Medicine, Ghent University (Belgium). Sixteen CD-1 lactating female mice (Harlan Laboratories Inc., Horst, The Netherlands) were used 12-14 days after birth of the offspring. The pups were removed 1 to 2 h before bacterial inoculation of mammary glands and a mixture of ketamine/xylazine was used for anesthesia of the lactating mice. The orifice of both L4 (on the left) and R4 (on the right) abdominal mammary glands was exposed by a small cut at the near end of the teat. 100 μL PBS without (*n *= 2) or with 150 CFU of *S. aureus *strain O11 (*n *= 7) or O46 (*n *= 7) was injected slowly with a 32-gauge blunt needle through the teat canal. Rectal body temperature of the mice was measured at 0 h and 18 h pi. At 18 h pi mice were anesthetized with ketamine/xylazine to collect blood by cardiac puncture and serum was obtained after clotting at 37 °C and cold centrifugation. After cardiac puncture, mice were euthanized by cervical dislocation and mammary glands were isolated. Glands of six mice of each group were homogenated and bacterial CFU was quantified by plating serial logarithmic dilutions in PBS. Lysates from the homogenates were prepared in 1% NP40-based buffer. Serum and mammary gland lysates were quantified for IL-1β, IL-6, TNF, KC and MCP-1 using BD^™ ^Cytometric Bead Array technology. Mammary glands (inoculated and PBS control glands) of 4 mice (2 from each group) were embedded and used for further histopathological analysis (Vetopath, Antibes, France).

### Statistical analyses

A Fisher test was used with a risk α = 10% to determine the difference between the ewes infected with *S. aureus *O11 and the ewes infected with strain O46. Differences in rectal body temperature and cytokine levels in the mouse mastitis model were analyzed with the unpaired *T*-test. *P *< 0.05 was considered statistically significant.

## Results

### Ewes infected with O11 or O46 *S. aureus *strains developed mastitis with different severities

Although O11 and O46 strains share the same genotype and are highly genetically similar [10], they were isolated from dramatically different ewe mastitis episodes. One can thus wonder whether the clinical signs associated with O11 and O46 infection were or not related to strains characteristics or a fortuitous matter of sampling time (mastitis can indeed evolve from subclinical to severe clinical or even gangrenous within a few days). To check this, two groups of ewes were infected either with O11 or O46 *S. aureus *strains, as described in the previous section. Onset of the symptoms was followed up during the course of the experiment. All animals became infected and signs of mastitis were evident in most ewes as soon as 24 h pi. The animals shed the *S. aureus *strains over the sampling period and remained infected for the duration of the experiment. Shedding from the infected glands varied and *S. aureus *load in milk ranged from 10 CFU/mL to 3.16 × 10^8 ^CFU/mL, depending on the individual ewe, and on the day pi (not shown). Symptoms evoked by intramammary inoculation varied among ewes. In group O11, five out of six ewes developed a gangrenous mastitis, the last one developed a pyogenic mastitis according the criteria defined in Additional file 1, Table S1. In group O46, symptoms were more heterogeneous and mastitis cases were classified in subclinical mastitis (*n *= 1), pyogenic mastitis (*n *= 2), mild clinical mastitis (*n *= 2) and gangrenous mastitis (*n *= 1). Subclinical mastitis was determined with the presence of bacteria (250 CFU/mL of milk 36 h pi), a raise in SCC (> 200 000 cells/mL in each milk sample after 24 h) and absence of fever or symptoms. Except for the ewe with subclinical mastitis, bacteria were detected in all milk samples and reached more than 10^6 ^CFU/mL 36 h pi. All animals had fever (above 40 °C 36 h pi) and SCC increased quickly and was above 10^6 ^cells/mL at 36 h pi. The proportion of the gangrenous mastitis was significantly higher in the group of ewes infected with O11 strain compared to the group infected with O46 (*p *= 0.08) (Additional file 2, Figure S1).

*S. aureus *O11 and O46 induce dramatically different clinical features in infected mice. To confirm our observation that *S. aureus *strains O11 or O46 induce different types of mastitis, the mouse mastitis model was employed in the current study. Strains O11 and O46 grew equally well in the infected mouse mammary glands and induced mastitis, as determined by temperature measurement (hypothermia in O11 group and hyperthermia in O46 group, 24 h pi; Additional file 3, Figure S2) and histopathological analysis: polymorphonuclear neutrophils (PMN) infiltration was observed only in infected (either with O11 or O46 strains) mammary gland tissue (not shown). To analyse the role of each strain in the development of mastitis, cytokine profile of the serum and mammary gland tissue lysates of mice infected with O11 strain was compared to those infected with O46 strain. The results of cytokine quantification showed that mice infected with *S. aureus *O46 had significantly higher IL-1β and TNF levels in the mammary gland lysates and significantly higher systemic (serum) levels of IL-1β and MCP-1 (Additional files 4 and 5, Figures S3 and S4). Altogether, these results demonstrate that despite their close genetic relationships, *S. aureus *O11 and O46 reproducibly induced mastitis with significantly different clinical signs.

### Antibody production in response to infection of ewes with *S. aureus *strains O11 or O46

**Figure 1:**
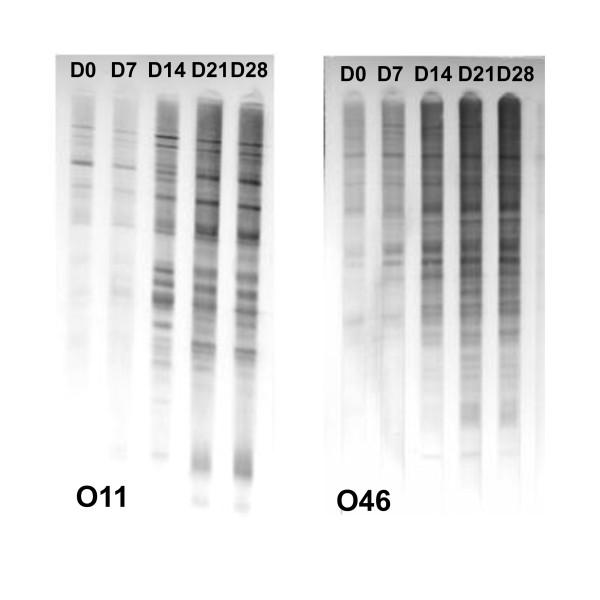
**Western blot analysis of total lysates of *S. aureus *O11 (left panel) or *S. aureus *O46 (right panel) using D0, D7, D14, D21, D28 diluted 1:10,000 sera of ewes infected by O11 and O46**. Bacteria were grown in iron-depleted RPMI without aeration to late exponential phase. Protein samples were prepared from total cell, submitted to 1-DE, western blotted on PVDF membrane and revealed using each of the 6 serum samples collected on ewes infected in each group. One representative example is given for each group of infected ewes (with O11 or O46).

To compare the relative level of antibodies developed in response to *S. aureus *presence in the mammary gland, serum sampled on D0, D7, D14, D21 and D28 pi were analysed by Western blotting using either O46 or O11 total bacterial extracts as described in the previous section. The number of bacterial proteins recognised by sera and signal intensity increased from D0 to D28 for both O11 and O46 samples (Figure 1). The intensity of the signal revealed with sera collected on D0 was low and much weaker compared to those obtained with sera collected on D21 or D28. Western blots membranes were analysed as described in the previous section. Sera collected either on D21 or D28 were selected for further analysis. Sera yielding the best signals in each group (one sample for each of the six ewes) were pooled to be used in SERPA experiments. Two pools were thus obtained: sera from ewes infected with O11 and sera from ewes infected by O46, hereafter referred to as group O11 sera and group O46 sera, respectively.

### Detection and identification of immunogenic staphylococcal proteins by SERPA

**Figure 2:**
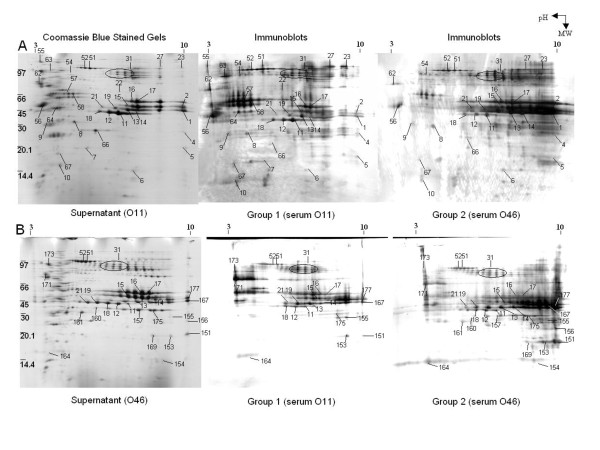
**Representative 2-DE gels and SERPA on supernatant fractions of *S. aureus *O11 (A) and *S. aureus *O46 (B)**. Supernatant samples were prepared from stationary phase cultures of *S. aureus *strains grown anaerobically on iron-depleted RPMI. Preparative 2-DE gels were Coomassie blue stained (left panel). Gels run in parallel were immunoblotted using the pools of sera obtained from group 1 (infected with O11) animals (middle panels) or from group 2 (infected with O46) animals (right panels). Samples were run in parallel on 13 cm gels (pI 3-10; 14% SDS-PAGE). Spots identified by MS/MS are labeled.

**Figure 3:**
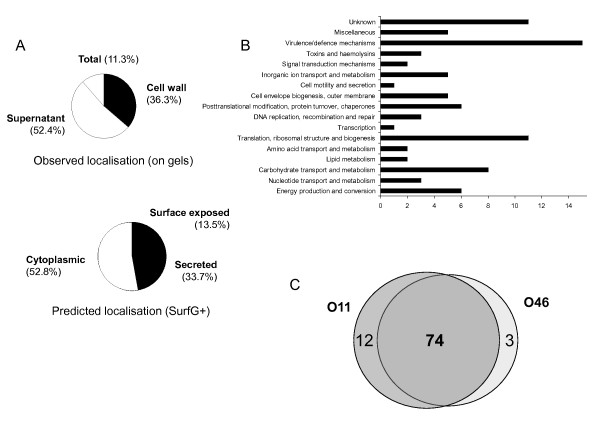
**(**A**) Distribution of the immunoreactive proteins based on their experimentally observed (upper panel) or predicted (lower panel) subcellular localization**. (**B**) Distribution of the immunoreactive proteins based on their GO functional classes. (**C**) Venn diagram of immunoreactive proteins defining the core and the accessory seroproteomes.

Protein samples were prepared from O11 and O46 strains grown in conditions that best mimic the mastitis context [11]. Each fraction (total, cell wall and supernatant) of O11 and O46 culture was immunoblotted using either group O11 or group O46 sera. Altogether, 89 proteins were identified as immunoreactive (Table 2). Comparison of SERPA results (see Figure 2, and Additional files 6 and 7, Figures S5 and S6) on the three fractions analyzed showed that immunoreactive proteins were mainly identified in supernatant samples prepared from aerobic and anaerobic cultures (Figure 2 and Additional file 7, Figure S6) and cell wall samples (Additional file 6, Figure S5, upper panels) whereas total protein samples were poorly recognized (Additional file 6, Figure S5, lower panels). A vast majority of the immunoreactive proteins are thus found in supernatant and cell wall fractions (88.7%), and only 11.3% are found in the total fractions (Figure 3A). Of note, secreted and surface proteins are expected to be exposed to the host immune system. The predicted location of the proteins (according to the SurfG+ analysis of O11 and O46 genome sequences) [23], showed that the immunoreactive proteins were mainly predicted cytoplasmic (52.8%) (Figure 3A). Proteins were classified into categories based on functional annotation (Figure 3B). Most immunogenic proteins identified here are found in various functional categories involved in cellular machinery and metabolism; while 20% were virulence factors and virulence associated proteins.

**Table 2:** Proteins identified in this study.

Description^1^	Spots^2^	O11 CDS^3^	O46 CDS^3^	ED133 CDS^3^	pI^4^	Mass (Da)^5^	Score^6^	Cov.^7^	#pep.^8^	EmPAI^9^	Comp.^10^	Loc.^11^	Immun.^12^
**Metabolism**													

***Energy production and conversion***													

formate acetyltransferase	81, 83, 82	011_0041	046_0511	SAOV_0163	5,31	84808	1301,60	34,31	22	1,57	CW	C	

ldh L-lactate dehydrogenase	82, 99, 81, 83, 95	011_0136	046_0198	SAOV_2646c	4,80	34389	1749,37	63,64	26	21,75	CW	C	[82]

**L-LACTATE DEHYDROGENASE (O11)**	**64**	**011_0021**	**046_0531**	**SAOV_0178**	**5,00**	**34548**	**745,83**	**50,79**	**14**	**2,59**	**S**	**C**	[82]

bifunctional acetaldehyde-CoA/alcohol dehydrogenase	79	011_0796	046_0709	SAOV_0095	5,68	94945	2090,64	45,45	33	2,26	CW	C	

D-3-phosphoglycerate dehydrogenase	100	011_0585	046_1131	SAOV_2344	5,32	34652	717,85	43,53	11	1,72	CW	C	

atpD F0F1 ATP synthase subunit beta	62, 90	011_2064	046_0898	SAOV_2144c	4,68	51368	631,03	30,21	10	0,98	S,CW	C	[83]

***Nucleotide transport and metabolism***													

adk adenylate kinase	9	011_0510	046_1206	SAOV_2266c	4,80	23375	531,72	42,38	9	2,80	S	C	[84]

deoD purine nucleoside phosphorylase	9	011_1051	046_0577	SAOV_2178	4,85	25892	470,94	53,39	8	1,97	S	C	[4]

guaB inositol-monophosphate dehydrogenase	102, 129	011_0077	046_0960	SAOV_0412	5,61	52818	1237,95	54,30	20	3,52	CW,T	C	[4]

***Carbohydrate transport and metabolism***													

gpmA 2,3-bisphosphoglycerate-dependent phosphoglycerate mutase	131	011_1952	046_0264	SAOV_2463c	5,23	26663	271.65	30.26	5	0.80	S,T	C	

eno enolase 2-phosphoglycerate dehydratase	62, 88, 90, 96, 137	011_2336	046_2241	SAOV_0818	4,55	47088	1868,52	63,59	25	7,09	S,CW,T	C	[4,52,85-87]

fda fructose-1,6-bisphosphate aldolase	107, 94, 72, 91, 95	011_0131	046_0202	SAOV_2650	5,06	32878	852,09	46,62	13	2,48	CW,S	C	[85-88]

gap glyceraldehyde-3-phosphate dehydrogenase	99, 57, 81, 82, 83	011_2340	046_2237	SAOV_0814	4,89	36258	940,06	46,13	13	3,04	CW,S	C	[82,86,87,89,90]

putative translaldolase	9	011_1872	046_2451	SAOV_1765	4,72	25689	554,11	46,84	10	3,32	S	C	

tpiA triosephosphate isomerase	9	011_2338	046_2239	SAOV_0816	4,81	27271	1396,16	76,28	21	16,78	S	C	[4,91,92]

atl autolysin	23, 31	011_1991	046_0320	SAOV_0999c	9,59	136983	2567,68	42,14	42	1,87	S	S	[52,76,93]

pyk pyruvate kinase	105	011_1282	046_0580	SAOV_1685	5,23	63067	271,45	11,62	5	0,29	CW	C	[94]

***Lipid metabolism***													

fabZ 3R-hydroxymyristoyl ACP dehydratase	126	011_2356	046_1839	SAOV_2140c	5,71	16071	86,65	13,70	2	0,47	T	C	

acetoin reductase	106	011_1332	046_1393	SAOV_0074	5,04	27199	566,84	40,31	7	1,82	CW	C	

***Amino acid transport and metabolism***													

dipeptidase PepV	62	011_1904	046_1329	SAOV_1737	4,56	52762	235.33	10.66	4	0,27	S	C	

**CYSTEINE SYNTHASE (O11)**	**64**	**011_1382**	**046_2402**	**SAOV_0535**	**5,38**	**32955**	**266.80**	**28.71**	**5**	**0,61**	**S**	**C**	

**Information storage and processing**													

***Translation, ribosomal structure and biogenesis***													

fus elongation factor G	95	011_0401	046_1769	SAOV_0582	4,80	76564	2173,77	68,11	30	4,11	CW	C	[3,90]

prs 50S ribosomal protein L25/general stress protein Ctc	94	011_1370	046_2414	SAOV_0523	4,39	23773	384,17	34,56	6	2,26	CW,S	C	[95]

rplC 50S ribosomal protein L3	131	011_0531	046_1185	SAOV_2287c	9,72	22648	482,78	42,11	9	2,95	T	C	[95]

rpsA 30S ribosomal protein S1	137, 62, 121, 171	011_2142	046_1743	SAOV_1482	4,51	43252	1555,46	71,36	23	6,25	S,CW,T	C	[39,95]

rpsD 30S ribosomal protein S4	139	011_1260	046_1365	SAOV_1706	10,02	22999	428,22	40,50	8	1,96	T	C	[95]

rplB 50S ribosomal protein L2	91	011_0528	046_1188	SAOV_2284c	10,77	30136	239,93	25,27	5	0,69	CW	C	[95]

tsf elongation factor Ts	100, 64	011_0909	046_0755	SAOV_1259	5,05	32474	436,02	31,06	7	0,97	CW,S	C	[4,86,93,96]

tuf elongation factor Tu	90, 88, 96, 10, 122, 137, 149, 81, 82, 83, 95	011_0402	046_1768	SAOV_0583	4,74	43077	2665,48	84,26	38	52,14	S,CW,T	C	[3,52,82,85,86,90,93,96]

yfiA ribosomal subunit interface protein	106	011_2483	046_1255	SAOV_0789	5,25	21511	376,75	35,33	6	1,76	CW	C	

aspS aspartyl-tRNA synthetase	63	011_2454	046_2303	SAOV_1627	4,99	66584	233,90	7,65	4	0,21	S	C	

alaS alanyl-tRNA synthetase	82	011_2466	046_2291	SAOV_1616	5,00	98604	442,31	11,74	8	0,30	O	C	[97]

***Transcription***													

nusA transcription elongation factor NusA	62	011_0919	046_0765	SAOV_1268	4,60	43701	247,74	11,00	4	0,34	S	C	

***DNA replication, recombination and repair***													

dnaN DNA polymerase III subunit beta	90	011_1166	046_1471	SAOV_0002	4,66	41888	706,28	45,62	11	1,30	CW	C	

nuc staphylococcal thermonuclease precursor	151, 108, 5, 153, 206, 207, 217	011_2070	046_2528	SAOV_0832	9,20	25089	967,00	50,00	20	14,46	S,CW	PSE	[98]

ruvA Holliday junction DNA helicase	66	011_2442	046_2315	SAOV_1639	5,77	22249	137,72	17,00	3	0,52	S	C	

***Posttranslational modification, protein turnover, chaperones***													

ahpC alkyl hydroperoxide reductase subunit C	67, 82, 83, 99, 95	011_0085	046_0968	SAOV_0404c	4,88	20963	667,36	56,08	11	4,95	S,CW	C	[93]

**AhpF ALKYL HYDROPEROXIDE REDUCTASE SUBUNIT F (O11)**	**97, 86**	**011_0086**	**046_0969**	**SAOV_0403c**	**4,68**	**54655**	**2242,14**	**67,06**	**34**	**9,91**	**CW**	**C**	

dnaK chaperone protein	96, 173, 137	011_2230	046_2216	SAOV_1580	4,63	46021	2907,83	80,29	43	24,66	S,CW,T	C	[90,96,99]

peptidyl-prolyl cis-isomerase	212, 1, 68, 91	011_2089	046_2477	SAOV_1837	9,01	35602	574,32	31,56	11	1,66	S,CW	PSE	

tig trigger factor	105	011_1304	046_0602	SAOV_1664	4,34	48565	1061,87	61,43	17	2,25	CW	C	[94]

**TrxB THIOREDOXIN REDUCTASE (O11)**	**64**	**011_2580**	**046_2228**	**SAOV_0801**	**5,21**	**33595**	**681,95**	**33,76**	**11**	**1,81**	**S**	**C**	[93]

**Cellular processes**													

***Cell envelope biogenesis, outer membrane***													

**IsaA IMMUNODOMINANT ANTIGEN A (O46)**	**185, 8, 161, 186, 9, 164, 189**	**011_0168**	**046_0166**	**SAOV_2614c**	**5,91**	**24219**	**1516,56**	**59,23**	**22**	**40,99**	**S**	**S**	[3,75]

isdA iron-regulated cell wall-anchored protein	31, 27, 73, 74, 75, 83, 118, 79, 81, 82, 95	011_1476	046_1296	SAOV_1125c	8,69	70445	2288,12	57,87	38	6,04	S,CW	PSE	[100-102]

isdB cell surface transferrin-binding protein	212, 211, 210, 208, 193, 156, 138, 110, 70, 86, 131, 156, 193	011_1477	046_1295	SAOV_1126c	9,54	39197	886,30	45,48	15	4,45	S,CW,T	PSE	[54,100,103]

**IRON-REGULATED SURFACE DETERMINANT PROTEIN H (O11)**	**55, 31**	**011_1248**	**046_1353**	**SAOV_1717**	**5,05**	**100650**	**2209,72**	**42,11**	**37**	**2,58**	**S**	**PSE**	[75]

IsdD iron-regulated protein	15, 16	011_1480	046_1292	SAOV_1128	8,51	41357	278,05	15,36	5	0,47	S	PSE	

***Cell motility and secretion***													

N-acetylmuramoyl-L-alanine amidase	52, 51, 187	011_1090	046_1546	SAOV_2693	5,87	69226	3097,93	71,57	43	16,52	S	S	[57,75,76]

***Inorganic ion transport and metabolism***													

nasE assimilatory nitrite reductase	10	011_1932	046_0245	SAOV_2445c	4,95	11430	126,05	23,08	2	0,70	S	C	

mntC Manganese/iron transport system substrate-binding protein	94	011_2274	046_0062	SAOV_0666c	8,68	34719	1183,08	51,46	20	10,68	S,CW	PSE	

sirA iron-regulated lipoprotein	135, 64	011_1345	046_1405	SAOV_0062	9,20	36735	609,79	35,45	11	1,58	S,T	PSE	[104]

fhuD2 ferrichrome-binding protein	91	011_0566	046_1150	SAOV_2323c	9,16	33990	406,75	30,13	8	1,10	CW	PSE	[105]

ferrichrome ABC transporter lipoprotein	91	011_1857	046_1674	SAOV_2224c	9,44	36751	600,45	38,41	11	1,57	CW	PSE	

***Signal transduction mechanisms***													

SA1540 GAF domain-containing protein	10	011_1261	046_1366	SAOV_1705	5,09	17042	139,34	22,73	3	0,72	S	C	

Universal stress response protein	7, 123	011_1269	046_1374	SAOV_1697	5,60	18463	973,68	78,31	12	19,34	S,CW	C	[106]

**Toxins and haemolysins**													

beta-hemolysin	15, 19, 205	011_1750	046_2394	SAOV_2040	8,75	37386	1308,79	61,03	20	4,92	S	S	[57,107]

hla alpha-hemolysin precursor	13, 1, 15, 19, 21, 68, 72, 107, 146, 153	011_1514	046_1259	SAOV_1161c	8,87	36329	2238,72	76,71	34	44,90	S,CW,T	S	[93,107]

hlgC gamma-hemolysin component C	68, 1	011_1956	046_0268	SAOV_2469	9,29	35562	765,07	31,43	12	1,90	S,CW	S	[57]

**Virulence/defence mechanisms**													

**Sbi IGG-BINDING PROTEIN SBI (O11)**	**216, 217, 212**	**011_1954**	**046_0266**	**SAOV_2466**	**9,38**	**49998**	**984,16**	**41,74**	**18**	**2,80**	**S**	**S**	[76]

**SspB CYSTEINE PROTEASE PRECURSOR (O11)**	**58, 57, 64, 182, 215**	**011_2154**	**046_0327**	**SAOV_0993c**	**5,45**	**42714**	**2027,86**	**65,52**	**30**	**11,52**	**S**	**S**	[4]

SplF serine proteinase	4	011_0672	046_2496	SAOV_1795	9,36	25638	255,88	23,01	5	0,84	S	S	[108]

lukD leukotoxin D subunit	1	011_0685	046_2484	SAOV_1812	9,14	36936	365,37	22,02	7	0,98	S	S	[109]

lukE leukotoxin E subunit	1, 68	011_0686	046_2483	SAOV_1813c	9,38	34126	451,11	17,65	7	1,77	S,CW	S	[109]

leukocidin chain lukM precursor	68, 1, 120, 145, 177, 194, 201, 202, 94	011_1215	046_2777	SAOV_1909	9,41	35054	2504,69	71,43	35	29,80	S,CW,T	S	[58,110]

leukocidin F subunit	16, 70	011_1752	046_1972	SAOV_2041	8,29	38639	1154,65	45,27	19	11,64	S,CW	S	[58,110]

leukocidin S subunit	199, 5, 70, 108, 109, 197, 200, 211	011_1753	046_1973	SAOV_2042	9,38	40379	1399,91	55,56	23	6,70	S,CW	S	[58,110]

Panton-Valentine leukocidin LukF-PV chain precursor	1, 68, 70, 15, 31, 119, 195, 203, 204, 10, 91	011_1216	046_2776	SAOV_1908	9,16	36496	965,38	50,31	15	3,37	S,CW	S	[58,110]

plc 1-phosphatidylinositol phosphodiesterase	11, 12, 19	011_1424	046_1419	SAOV_0049	7,12	37030	2759,20	71,95	44	84,00	S	S	[4]

**Aur ZINC METALLOPROTEINASE AUREOLYSIN (O11)**	**220, 63**	**011_1083**	**046_1553**	**SAOV_2686c**	**4,98**	**54947**	**921,93**	**47,39**	**17**	**1,68**	**S**	**C**	[76,111]

**Opp1A OLIGOPEPTIDE TRANSPORTER SUBSTRATE BINDING PROTEIN (O11)**	**86**	**011_2424**	**046_2102**	**SAOV_2517c**	**8,33**	**59224**	**1398,01**	**47,53**	**24**	**3,28**	**CW**	**PSE**	

**SspA GLUTAMYL ENDOPEPTIDASE SERINE PROTEASE (O11)**	**56, 184, 214**	**011_2155**	**046_0325**	**SAOV_0994c**	**4,68**	**32250**	**1575,55**	**69,26**	**24**	**24,18**	**S**	**C**	[57]

**SECRETED VON WILLEBRAND FACTOR-BINDING PROTEIN (O11)**	**22**	**011_2679**	**046_0987**	**SAOV_2051c**	**8,39**	**57935**	**1122,89**	**36,67**	**17**	**1,70**	**S**	**S**	

epidermal cell differentiation inhibitor B	208, 209, 193, 156, 4	011_0489	046_2741		9,51	27969	1385,18	69,32	22	22,31	S	S	

**Miscellaneous**													

adhA alcohol dehydrogenase	100	011_1554	046_0086	SAOV_0640	5,34	36025	1167,82	66,37	16	4,30	CW	C	[87,93]

exported secretory antigen precursor	67	011_0584	046_1132	SAOV_2343	5,77	17388	397,17	33,13	5	1,43	S	S	

lip triacylglycerol lipase precursor	31, 27	011_1119	046_1518	SAOV_2721c	8,13	76637	1723,17	50,95	29	2,36	S	S	[93]

mqo malate:quinone oxidoreductase	128	011_0130	046_0203	SAOV_2651c	6,12	55964	1047,99	46,79	17	2,50	T	C	

putative staphylococcal enterotoxin	151	011_0097	046_0981	SAOV_0394	9,54	23311	173,20	18,72	4	0,71	S	S	

**Unknown**													

**HYPOTHETICAL PROTEIN (O11)**	**220**	**011_0736**	**046_1969**	**SAOV_0454**	**4,89**	**56229**	**823,11**	**39,40**	**14**	**1,21**	**S**	**S**	

hypothetical protein (Similar to truncated map-w protein (91%))	88, 10, 62	011_1749	046_2393		9,91	53686	899,64	34,52	18	2,09	S,CW	S	[112]

hypothetical protein (Similar to beta-lactamase (84%))	10	011_0679	046_2490		6,84	20800	726,52	47,15	10	7,09	S	S	

**HYPOTHETICAL PROTEIN (O46)**	**157, 160, 175, 189, 193**	**011_0490**	**046_2740**		**8,88**	**30854**	**2343,27**	**74,29**	**41**	**65,07**	**S**	**S**	

hypothetical protein (Similar to probable glutamyl-endopeptidase (76%))	66	011_0488	046_2742		5,44	20552	741,93	47,62	12	5,11	S	C	

**SA0570 HYPOTHETICAL PROTEIN (O46)**	**219,154, 127**	**011_2290**	**046_0078**	**SAOV_0649**	**9,17**	**18554**	**815,59**	**69,05**	**13**	**15,93**	**S,CW**	**S**	

SA0663 hypothetical protein	10, 164, 94	011_2561	046_2636	SAOV_0742c	9,15	16047	457,11	35,62	8	3,63	S	PSE	

SA0914 hypothetical protein	6	011_2000	046_0311	SAOV_1008c	6,55	11338	522,48	57,14	8	13,10	S	S	

pathogenicity island protein	55	011_2741	046_0985	SAOV_0429	9,55	12071	204,61	19,64	3	1,12	S	S	

SA1607 hypothetical protein	91	011_1867	046_2456	SAOV_1770	6,04	34973	232,41	16,56	4	0,43	CW	S	

SA1402 hypothetical protein	91	011_2223	046_2209	SAOV_1573	5,60	35160	448,93	28,27	6	0,72	CW	S	

Gene products of the accessory seroproteome are indicated in bold capital letters, (O11) indicates O11-specific proteins, (O46) indicates O46-specific proteins.

All the other proteins belong to the core seroproteome

^1^: Proteins are classified in Gene Ontology functional classes. Names are given according to annotation of available *S. aureus *sequence genomes.

^2^: Spot number (see figures)

^3^: Coding sequence numbers corresponding to the identified proteins in *S. aureus *O11, *S. aureus *O46, and ED133, respectively.

^4^: Theoretical isoelectric point as determined from the predicted protein sequence

^5^: Theoretical Mass as determined from the predicted protein sequence

^6^: Mascot standard score

^7^: % of the protein sequence covered by the peptides identified

^8^: number of peptides identified

^9^: exponentially modified protein abundance index

^10^: Subproteome compartment where the spot was identified. CW, cell wall; S, supernatant; T, total fraction

^11^: predicted protein localization. C, cytoplasmic; S, secreted; PSE, predicted surface exposed

^12^: reported as immunogen elsewhere

### Composition of the core and accessory seroproteomes

SERPA revealed 74 proteins as being recognised by both group O11 and group O46 sera (Table 2). These proteins defined the core seroproteome, i.e. a pool of staphylococcal proteins that are recognized by the host immune system in both *S. aureus *strains (Figure 3C). Moreover, 15 proteins were differentially recognized by group O11 sera or by group O46 sera. These proteins defined the accessory seroproteome, i.e. a pool of strain-specific proteins that are recognized by the host immune system. Among these 15 proteins, 12 appeared to be immunogenic only in infections with O11 (Table 2). These proteins include 7 virulence-associated proteins (Sbi, SspB, SspA, Aur, IsdH, Opp1A, and VWbp), 2 stress response proteins (AhpF, TrxB), 1 hypothetical protein (product of CDS O11_0736 or O46_1969 in O11 or O46), Ldh and a cysteine synthase. Of note, when considering the corresponding genes, all of these 12 genes are present and highly similar in O11 and O46 strains. Only *ahpF *and *vwbp *present 2 and 1 non-synonymous single nucleotide polymorphisms (NS-SNPs), respectively. These results suggest that O11 produce these 12 proteins in vivo in a mastitis context whereas O46 does not, or to a much lesser extent. Three proteins appeared to be immunoreactive with group O46 sera only. Two of them are hypothetical proteins corresponding to SAOV0649 (CDS 011_2290 and 046_0078, in O11 and O46, respectively) encoding a probable esterase or lipase, and a gene 011_0490/046_2740 with no homology in ED133 genome sequence. The third protein is IsaA, a virulence-associated immunodominant antigen A. The genes encoding IsaA and the probable esterase or lipase are highly homologous in O11 and O46 with only 1 synonymous SNP found when comparing O11_2290 and O46_0078. Interestingly, the CDS O11-0490 in O11 genome shares homology with O46_2740 in O46. However, sequence analysis reveals that O11-0490 corresponds to a truncated form of O46_2740. This gene encodes a predicted protein that presents 59% identity and 76% homology with exfoliative toxin D [24].

### Selected *S. aureus *immunoreactive proteins are widely distributed among a panel of strains isolated from clinical vs. subclinical ewe mastitis

**Figure 4:**
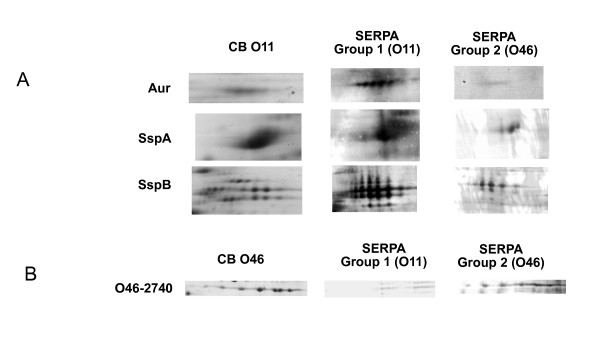
**SERPA of O11-specific (**A**) and O46-specific (**B**) immunoreactive proteins**. Protein specific signals were zoomed in on Coomassie blue stained gels (CB) and membranes revealed using sera obtained from animals infected with O11 (Group1) or O46 (Group 2). Aur, aureolysin; SspA, glutamyl endopeptidase serine protease; SspB, staphopain B; O46-2740, gene product similar to exfoliative toxin family.

**Figure 5:**
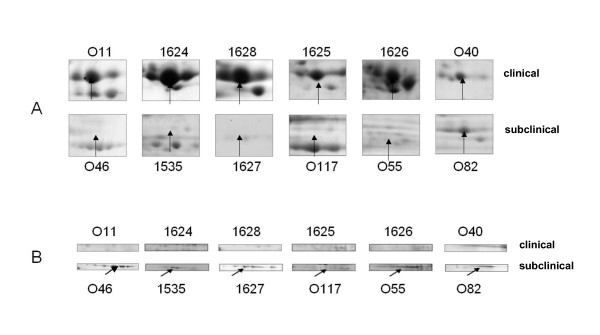
**Distribution of SspB (**A**) and O46-2740 product (**B**) in a panel of *S. aureus *strains isolated from clinical (a) and subclinical (b) mastitis**. Supernatant samples were prepared on stationary phase cultures of strains grown on iron-depleted RPMI. 2-DE gels were Coomassie blue stained.

In order to test the hypothesis that the seroproteomic variations identified by SERPA on the two *S. aureus *isolates were more widely distributed among isolates of a specific host- and clinical-association, we screened an additional 10 strains isolated from subclinical (*n *= 5) and severe (i.e. clinical or gangrenous mastitis; *n *= 5) cases of ewe mastitis for the presence of 2 selected proteins by proteome analysis of supernatant samples (i.e. 2-DE and Coomassie blue staining). The O11 (severe mastitis) protein selected was SspB, which belongs to a proteolytic cascade where a metalloprotease aureolysin (Aur) activates a serine protease zymogen proSspA, which in turn activates the SspB cysteine protease [25]. SspB and the two other proteins, SspA and Aur of the cascade, were recognized in O11 and yielded a very faint signal in O46 (Figure 4A). SspB, one element of this proteolytic cascade, was detected in the culture supernatant of strains isolated from clinical mastitis cases whereas it was not found in the strains isolated from subclinical mastitis (Figure 5A). The other protein tested was O46_2740 gene product (with similarity to exfoliative toxin family), which was specifically recognized in O46 infections and did not yield any significant signal in O11 infections (Figure 4B). When considering its presence in the culture supernatant of other *S. aureus *strains isolated from clinical or subclinical mastitis in ewes, we observed that it was only detected in the subclinical strains (100%) whereas it was undetectable in any of the strains isolated from clinical mastitis (Figure 5B). These results show that at least these tested proteins are differentially produced by *S. aureus *strains isolated from clinical or subclinical mastitis cases. Whether these differences are involved or not in the onset and the acuteness of the subclinical vs. clinical mastitis remains to be demonstrated.

## Discussion

Two closely related *S. aureus *ovine strains reproducibly induce distinct mastitis types in ewe and mouse models. Contrarily to *Escherichia coli *mastitis, which severity is mainly determined by host factors and not by the strains features [26], it seems that in *S. aureus*, inter-strain variations exist in terms of virulence potential. Such variations have been experimentally observed but the involved staphylococcal factors have not been clearly identified yet [27-29]. *S. aureus *content in virulence factors reportedly varies from strain to strain and this may account for the large panel of symptoms encountered in ruminant mastitis [30]. It was also shown that some elements of the core genome could account for host adaptation in *S. aureus *ruminant isolates [8]. Here, we show that two closely related *S. aureus *strains are able to induce dramatically different mastitis outcomes in animal model. The experimental mastitis induced in ewes confirmed that strain O11, originally isolated from a gangrenous mastitis, induced more severe infections than strain O46 in ewes. To corroborate the finding of the diversity of the clinical signs caused by O11 and O46, the mouse model of mastitis was employed for further investigation [31]. Despite variable susceptibility to viral, fungal or bacterial infections among the different mouse lines [32], the mouse model was validated as an experimental approach to the study of bovine mastitis [33]. In this study, CD-1 mice infected with O11 or O46 revealed diverse clinical signs and showed different cytokine profiles: O46 induced higher IL-1β and TNF-α levels in the mammary gland lysates and higher serum levels of IL-1β and MCP-1. It has been shown that the cytokines play a pivotal role in the development of the mastitis [34-36]. Recent investigation revealed that the proinflammatory cytokines, TNF-α and IL-1β, increased the capacity of bovine endothelial cells to eliminate intracellular *S. aureus *[37]. Endothelial cells may so represent a functional and active defence barrier against bacteria invasion of infected quarters. Higher synthesis of proinflammatory cytokines in O46-infected mice might thus reflect a higher anti-staphylococcal response and may allow more effective elimination of strain O46. Further investigation has to be conducted to confirm this hypothesis. Altogether, our findings demonstrated that these two well-characterized strains reproducibly induce drastically different mastitis in terms of severity, and will prove useful tools to gain further insights into host-pathogen interactions.

### Inventory of mastitis-associated core and accessory seroproteomes in *S. aureus *reveals new mastitis-related antigens

This study reports the application of SERPA to determine the core and accessory seroproteomes resulting from experimental ewe mastitis induced by two different *S. aureus *strains, isolated from a subclinical mastitis (O46) and a gangrenous mastitis (O11). To determine which proteins are recognized by the ewe immune system during mastitis with different severity, experimental infections were carried out on primiparous ewes using these two *S. aureus *strains. By comparison of the SERPA profiles, this study allowed, for the first time, the determination of core and accessory seroproteomes for *S. aureus *in a mastitis context. Eighty nine proteins were immunogenic in ewe mastitis implying they were produced during the infection. Seventy four were found in both seroproteome profiles and composed a core seroproteome. Fifteen of them were found strain-specific and composed the accessory seroproteome. Among the 74 proteins belonging to the core seroproteome, only 44 (59.5%) had already been reported to be immunogenic in infections caused by *S. aureus *or other pathogens (e.g. *Bacillus anthracis, Staphylococcus epidermidis, Clostridium perfringens*, *Schistosoma japonicum*) (Table 2). Among these previously described antigens, only 30S ribosomal S1 and LukM/LukF'-PV were previously reported to be immunogenic in *S. aureus *bovine mastitis [38,39]. These 44 proteins include most of the immunoreactive proteins categorized in Toxins-Haemolysins and Virulence/Defence mechanisms. The remaining 30 proteins (40.5%) are described for the first time as potential staphylococcal antigens in a mastitis context and, to our knowledge, were not described as immunogenic elsewhere. In the accessory seroproteome, 8 out the 15 proteins identified were already described as immunogenic elsewhere whereas 7 of them (AhpF, Opp1A, VVbp, Mqo, cysteine synthase and two hypothetical proteins) are described as such for the first time (Table 2). A majority of the proteins identified here are thus reported as immunogenic for the first time in a mastitis context. Furthermore, 37 out of 89 proteins of the total seroproteome (~42%) correspond to proteins described as staphylococcal antigens for the first time whatever the infection considered. Only four of these new antigens are categorized in Toxins-Haemolysins and Virulence/defense mechanisms. The other ones are mostly found in Metabolism (*n *= 9), Information storage (*n *= 7), and Unknown function (*n *= 13). Only a few studies previously analyzed *S. aureus *proteome and all of them were carried out on human isolates, which reportedly differ from ruminant isolates [8,9,40] and the strains were grown in laboratory culture conditions [4,41-46]. Here we used two ovine isolates grown in culture conditions mimicking the mastitis context [11]. These two criteria might account for the abundance of new staphylococcal antigens identified in this study.

### Importance of secreted and exported proteins in seroproteomic patterns

It is commonly assumed that surface exposed proteins play a role in host-pathogen interactions and that, because of their cellular localization, they are preferentially recognized by the host immune system. In this study, immunoreactive proteins were mainly identified in protein samples prepared from supernatant and cell wall fractions revealing, somehow, discrepancies between experimental (gels of total, cell wall and supernatant protein fractions) and theoretical localization (as determined in silico). Indeed, among the 47 immunoreactive proteins that were predicted cytoplasmic, only 10 were experimentally found in the total fraction and 37 were found in supernatant or cell wall fractions (Table 2). Such discrepancies were observed in other studies as well [4,47,48]. Some proteins are multifunctional and found both intra- and extracellularly. For example, glyceraldehyde-3-phosphate dehydrogenase [49] and enolase [50] were shown to be both cytoplasmic and surface exposed. In addition to their metabolic role in the cytoplasm, they play a role as adhesins when exposed on the bacterial surface. Furthermore, a new mechanism of protein exportation in Gram positive bacteria was recently described and not included yet in prediction tools for protein localization. It has actually been demonstrated that *S. aureus*, like some Gram negative bacteria, secretes membrane vesicles, which contained at least 90 different proteins [51]. Twelve of the proteins identified here were found in the vesicle-secreted proteins identified by Lee et al. [51]. Whether the other immunoreactive proteins identified here are secreted through a membrane vesicle mechanism remains undetermined.

### The core seroproteome

Proteins belonging to the core seroproteome are immunogenic regardless the severity of the induced mastitis. These proteins are therefore good targets for the development of new strategies against *S. aureus *mastitis. Some of them have been tested as vaccine target to prevent staphylococcal infections e.g., Enolase (Eno) [52], IsdA (an iron-regulated cell wall-anchored protein) and IsdB (a cell surface transferrin-binding protein) [53,54], GapC/B protein (glyceraldehyde-3-phosphate dehydrogenase) [55], Hla (alpha-hemolysin) [56]. These vaccines seem at least to limit infection damage (by notably decreasing mortality) but not to provide total effective protection.

Well-known virulence factors, such as Hla, Hlb (beta-hemolysin), SspA (V8 serine protease), ScpA (Staphopain A), and Plc (phosphatidylinositol phosphodiesterase), were identified here in a mastitis context and were previously identified as immunogenic in human infections [4,57]. Hla and leukotoxins were reportedly produced in vivo during mastitis [58] but to our knowledge this is the first time that the other proteins listed in Table 2 are shown to be produced in vivo during mastitis. These proteins deserve more attention to test their role in the mastitis onset and to check their potential use as target for vaccine development. Of note, we found 5 iron-related proteins (IsdA, IsdB, IsdH, MntC and SirA, 4 of which belonged to the core seroproteome) consistent with the culture conditions (iron depletion) and with a role in the physiologically important and difficult iron uptake in the mastitis conditions.

### The accessory seroproteome

Some proteins were shown to be specifically produced by strain O11 or by strain O46 in infected ewes. None of 12 proteins specifically produced by O11 in vivo had previously been reported as produced during mastitis. Their role in mastitis is thus unknown. Nevertheless most of them have been described as immunogenic in *S. aureus *infections in humans. Their function is mainly linked to resistance to host immune response like for IsdH [59], AhpF and TrxB, implied in oxidative stress responses [60] that may confer O11 resistance to neutrophils and so be an advantage compared to O46, Sbi that forms complexes with immunoglobulins Fc regions [61], Aur and SspB. Aureolysin is essential for activation of SspA [25], which in turn activates the SspB zymogen [62]. Aur, SspA and SspB seemed to be more produced in O11 than in O46. They can degrade conjunctive tissue [63]. Aureolysin has been shown to be involved in resistance to macrophage phagocytosis [64] and to significantly contribute to the activation of the fibrinolytic system [65]. It might thus reinforce the degradation of extracellular matrix in the mammary gland and promote bacterial spread and invasion. SspB can activate the chemoattractant chemerin, which results in a local inflammation of the tissue [66]. Moreover it induces the depletion of functional phagocytes at the site of infection by blocking phagocytosis by neutrophils and inhibiting their chemotactic activity [67]. SspB may so take part in the observed swelling of the mammary gland observed during gangrenous mastitis. Moreover, it has been shown that SspA and SspB play an important role in virulence in a mouse abscess model [68]. Opp1A was found to be produced by strain O11 in vivo. Opp proteins seem to take part in virulence in several infection models [69]. Although the role of Opp has not been clearly demonstrated until now, Opp proteins have also been reported to be involved in virulence in other Gram positive pathogens such as group A streptococci [70], *Streptococcus agalactiae *[71] or *Listeria monocytogenes *[72]. A variant of von Willebrand factor-binding protein gene has recently been located on the pathogenicity island SaPIov2, characteristic of small ruminant isolates [9]. Interestingly, besides being a coagulase, it is also an activator of pro-thrombin [73] that is present in cow milk [74]. Pro-thrombin activation in thrombin may have a pro-inflammatory effect during mastitis and thus take part in the symptoms observed in animals infected by O11. Whether and how these proteins are involved in the acuteness of the disease induced by O11 remains unknown. O46 specifically produced 3 immunoreactive proteins when compared to O11. IsaA has been reported to be immunogenic in many *S. aureus *infections [3,52,57,75-77]. It presents autolytic activity and is necessary for complete virulence [78] but its role in mastitis is not known. Interestingly, a protein (encoded by O46-2740) which shows similarity to exfoliative toxin D (ETD) was found produced by O46 and not by O11. Three amino-acids were shown to constitute the active site common to all the exfoliative toxins [79]. These amino-acids are present in O46_2740 gene (O46 strain) product but one is missing in O11-0490 gene product. The corresponding gene is not found in the recently released sequence of ovine strain ED133 [9] although we found its product in the exoproteome of 5 additional *S. aureus *strains isolated from subclinical mastitis. ETD is associated with cutaneous abscesses and furuncles [80]. Interestingly this toxin is also produced by Coagulase Negative Staphylococci species like *Staphylococcus hyicus*, *Staphylococcus pseudintermedius *and *Staphylococcus chromogenes*. CNS are highly prevalent in ovine subclinical mastitis [81]. Whether this particular toxin is specifically produced and plays a role during subclinical mastitis remains to be tested. Altogether, proteins differentially produced by O11 and O46 may be considered as potential marker of gangrenous or subclinical mastitis but this has still to be further demonstrated.

## Conclusion

To the best of our knowledge, this study provides the first comparative and comprehensive serological proteome analysis in a mastitis context. The proteins identified are immunogenic in ewes implying that they are also produced in the udder during infection. Many of them are found immunogenic for the first time and a great proportion was found in the supernatant and cell wall fractions even though they were predicted as cytoplasmic proteins. Whether or how these proteins are really involved in the mastitis infection and or the severity of the mastitis remains to be elucidated. This study provides a handful of interesting candidates for further investigations on their potential use as new targets for prophylactic or curative strategies such as vaccine or drug target as some appear to be involved in important virulence-associated functions (toxins, immune evasion, iron uptake).

## Competing interests

The authors declare that they have no competing interests.

## Authors' contributions

CLM participated in the experimental mastitis in ewes, carried out the 2-DE analyses, participated in the identification of the immunoreactive proteins and drafted the manuscript. JJ and GJ participated in the identification of the immunoreactive proteins by mass spectrometry, SE, NB and RT participated in the design of the study and in the results analyses, CP, JMG and EV carried out the experimental mastitis in ewes, DD and EM carried out the experimental mastitis in mice, DH, PF and JS carried out the genome sequencing and genome sequence analyses, EV and YLL conceived the study, and participated in its design and coordination. All authors read and approved the final manuscript.

## Supplementary Material

Additional file 1 **Table S1**: Criteria used to define the acuteness of mastitis symptoms Click here for file

Additional file 2 **Figure S1**: Dynamics of gangrenous mastitis onset in ewes infected by *S. aureus *O11 (argyles) and O46 (squares). Click here for file

Additional file 3 **Figure S2**: (**A**) Intramammary growth of *S. aureus *strain O11 and O46 in CD-1 mice. Populations are the mean values of *S. aureus *counts in homogenates of 12 mice mammary gland. (**B**) Temperature of infected mice 24h post-infusion. The mean value of the groups of mice infected with *S. aureus *O11 or O46 is given. The dashed line indicates the temperature of the animals at T0, before infection. Asterisks indicate statistically significant values. Click here for file

Additional file 4 **Figure S3**: Quantification of IL1β, IL6, TNF, KC and MCP-1 in mammary gland lysates with BD^™ ^Cytometric Bead Array. Cytokines were quantified on homogenates of mammary glands infected by *S. aureus *O11 or O46. Quantities are the mean values of 6 homogenates for each group (O11 and O46) and are expressed in pg/20 μg of total protein. Click here for file

Additional file 5 **Figure S4**: Quantification of IL1β, IL6, TNF, KC and MCP-1 in serum with BD^™ ^Cytometric Bead Array. Cytokines were quantified in sera collected on 12 mice infected by *S. aureus *O11 (6 sera) or O46 (6 sera). Quantities are the mean values of 6 sera for each group (O11 and O46) and are expressed in pg/mL. Click here for file

Additional file 6 **Figure S5**: Representative 2-DE gels and SERPA on cell wall fraction (upper panels) and total proteins (lower panels) of *S. aureus *O11. Supernatant samples were prepared from late exponential phase cultures of *S. aureus *strains grown anaerobically on iron-depleted RPMI. Preparative 2-DE gels were Coomassie blue stained (left panel). Gels run in parallel were immunoblotted using the pools of sera obtained from group 1 (infected with O11) animals (middle panels) or from group 2 (infected with O46) animals (right panels). Samples were run in parallel on 13 cm gels (pI 4-7; 12% SDS-PAGE). Spots identified by MS/MS are labeled. Click here for file

Additional file 7 **Figure S6**: Representative 2-DE gels and SERPA on supernatant fractions of *S. aureus *O46 (upper panels) and *S. aureus *O11 (lower panels). Supernatant samples were prepared from late exponential phase cultures of *S. aureus *strains grown aerobically on iron-depleted RPMI. Preparative 2-DE gels were Coomassie blue stained (left panel). Gels run in parallel were immunoblotted using the pools of sera obtained from group 1 (infected with O11) animals (middle panels) or from group 2 (infected with O46) animals (right panels). Samples were run in parallel on 13 cm gels (pI 3-10; 12% SDS-PAGE). Spots identified by MS/MS are labeled. Click here for file
