# Supplementary material for: Staphylococcus aureus seroproteomes discriminate ruminant isolates causing mild or severe mastitis
Source: Vet Res. 2011 Feb 15;42(1):35. doi: 10.1186/1297-9716-42-35 (PMC3052181; doi:10.1186/1297-9716-42-35)
Supplement: Additional file 1 — Table S1: Criteria used to define the acuteness of mastitis symptoms [file 1297-9716-42-35-S1.DOC]

**Table S1: Criteria used to define the acuteness of mastitis symptoms**

| **Severity** | **IDF definition*** | **SCC** | **Bacteria** | **Local symptoms** | **General symptoms** |
| --- | --- | --- | --- | --- | --- |
| **Gangrenous** |  | ↑↑ | "+" | The mammary gland becomes red then blue and black, is hot and then cold, the veins draining the gangrenous quarter are extensively thrombosed | Fast development, diarrhea, lameness, breath difficulties, dehydrated, fever, anorexia |
| **Severe clinical** | Udder inflammation characterized by sudden onset with grave systemic and local symptoms. This terminology is preferred to peracute clinical mastitis | ↑↑ | "+" | Half is swollen, red, hot and painful | Rapid pulse, depression, weakness and loss of appetite. |
| **Pyogenic** |  | ↑ | "+" | Presence of hard lumps, abscesses, or scars | No |
| **Mild clinical** | Observable abnormalities in milk. Generally clots or flakes with little or no signs of swelling of the mammary gland or systemic illness. | ↑ | "+" | A hot or sensitive udder may be slight or absent, however there may be signs of swelling. | No |
| **Subclinical** | Inflammation of the mammary gland that is not visible and requires a diagnostic test for detection. | ↑ | "+" | No | No |

*: after Group A2-International Dairy Federation: **Suggested Interpretation of Mastitis Terminology**. *Bull Int Dairy Fed* 1999, **338**:3-20.
