# Supplementary figures and images for: Staphylococcus aureus seroproteomes discriminate ruminant isolates causing mild or severe mastitis
Source: Vet Res. 2011 Feb 15;42(1):35. doi: 10.1186/1297-9716-42-35 (PMC3052181; doi:10.1186/1297-9716-42-35)

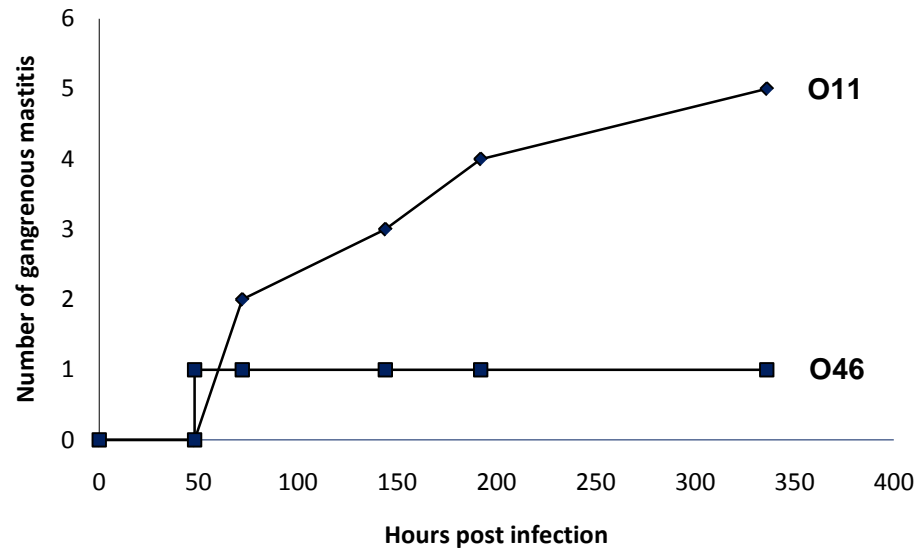

**Figure S1**

Supplement: Additional file 2 — Figure S1: Dynamics of gangrenous mastitis onset in ewes infected by S. aureus O11 (argyles) and O46 (squares). [file 1297-9716-42-35-S2.PDF]

**A**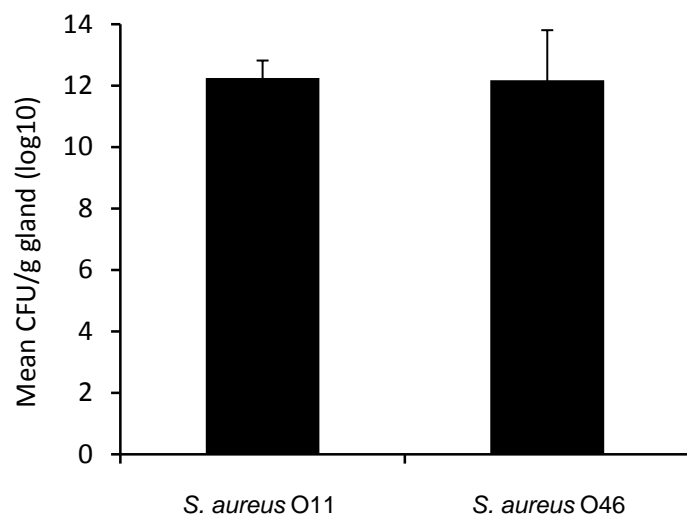**B**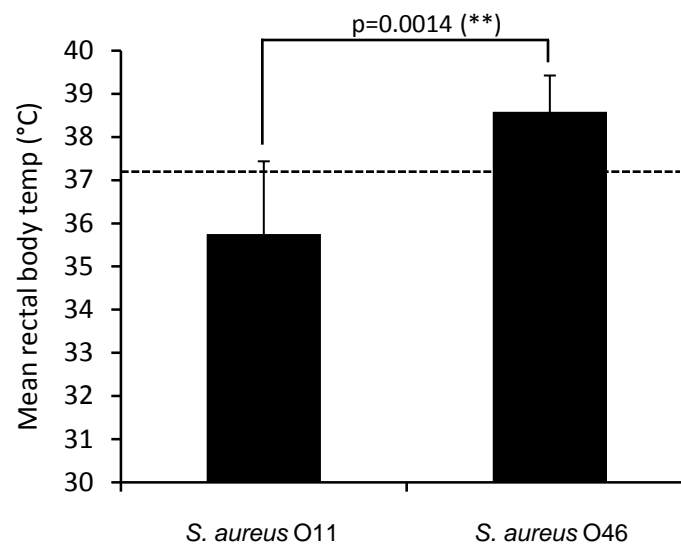**Figure S2**

Supplement: Additional file 3 — Figure S2: (A) Intramammary growth of S. aureus strain O11 and O46 in CD-1 mice. Populations are the mean values of S. aureus counts in homogenates of 12 mice mammary gland. (B) Temperature of infected mice 24h post-infusion. The mean value of the groups of mice infected with S. aureus O11 or O46 is given. The dashed line indicates the temperature of the animals at T0, before infection. Asterisks indicate statistically significant values. [file 1297-9716-42-35-S3.PDF]

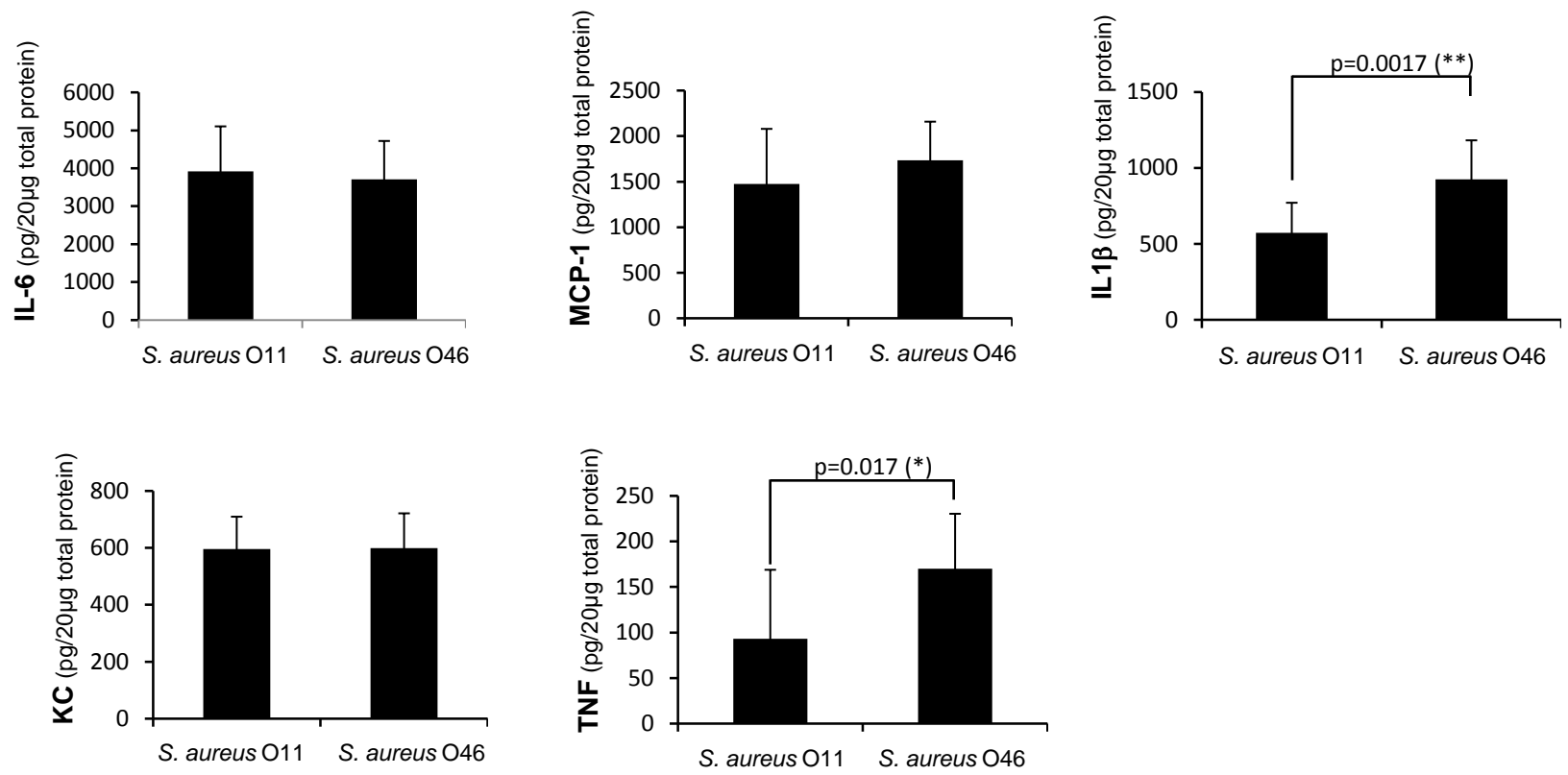

**Figure S3**

Supplement: Additional file 4 — Figure S3: Quantification of IL1β, IL6, TNF, KC and MCP-1 in mammary gland lysates with BD™ Cytometric Bead Array. Cytokines were quantified on homogenates of mammary glands infected by S. aureus O11 or O46. Quantities are the mean values of 6 homogenates for each group (O11 and O46) and are expressed in pg/20 μg of total protein. [file 1297-9716-42-35-S4.PDF]

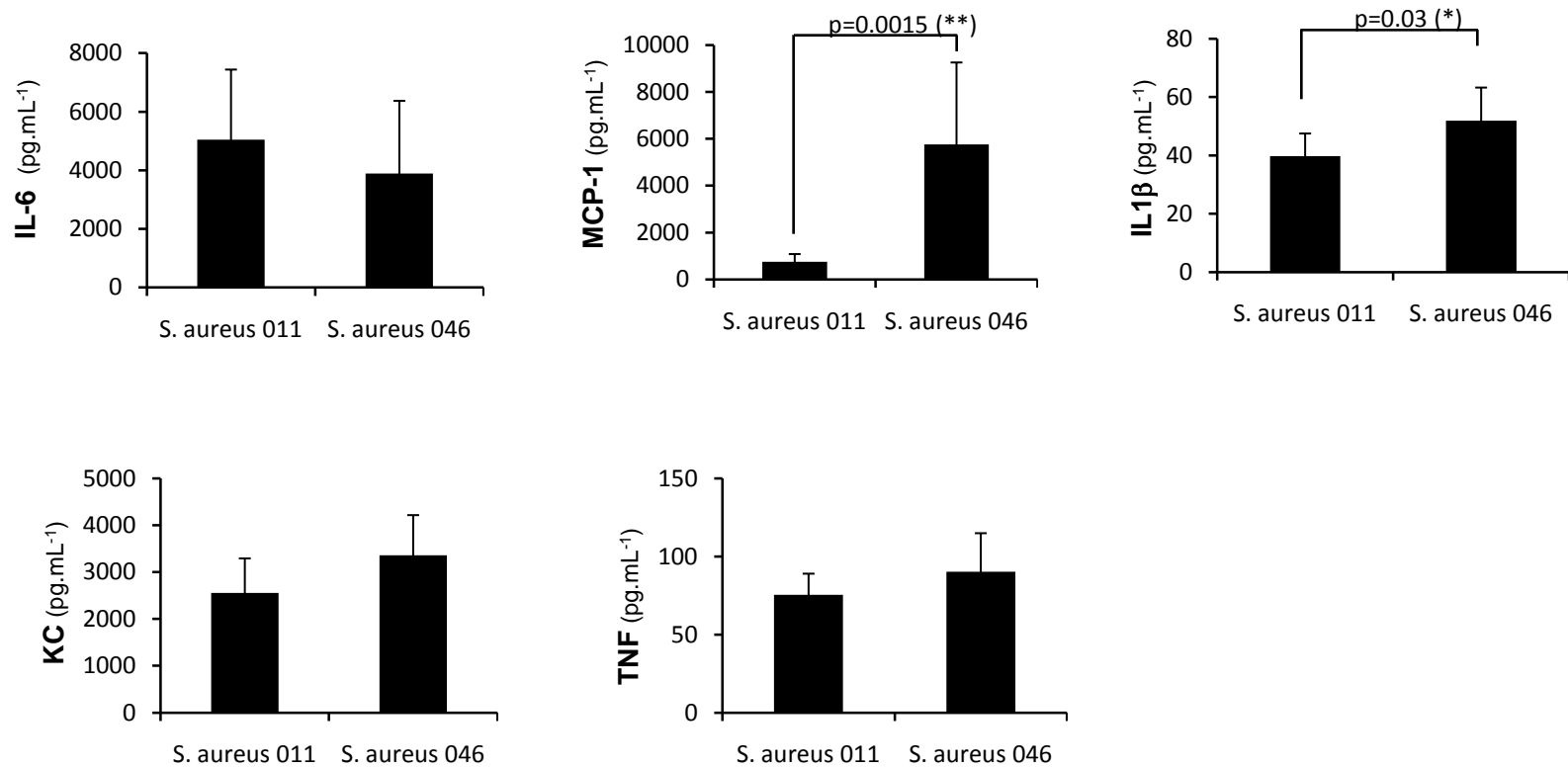

**Figure S4**

Supplement: Additional file 5 — Figure S4: Quantification of IL1β, IL6, TNF, KC and MCP-1 in serum with BD™ Cytometric Bead Array. Cytokines were quantified in sera collected on 12 mice infected by S. aureus O11 (6 sera) or O46 (6 sera). Quantities are the mean values of 6 sera for each group (O11 and O46) and are expressed in pg/mL. [file 1297-9716-42-35-S5.PDF]

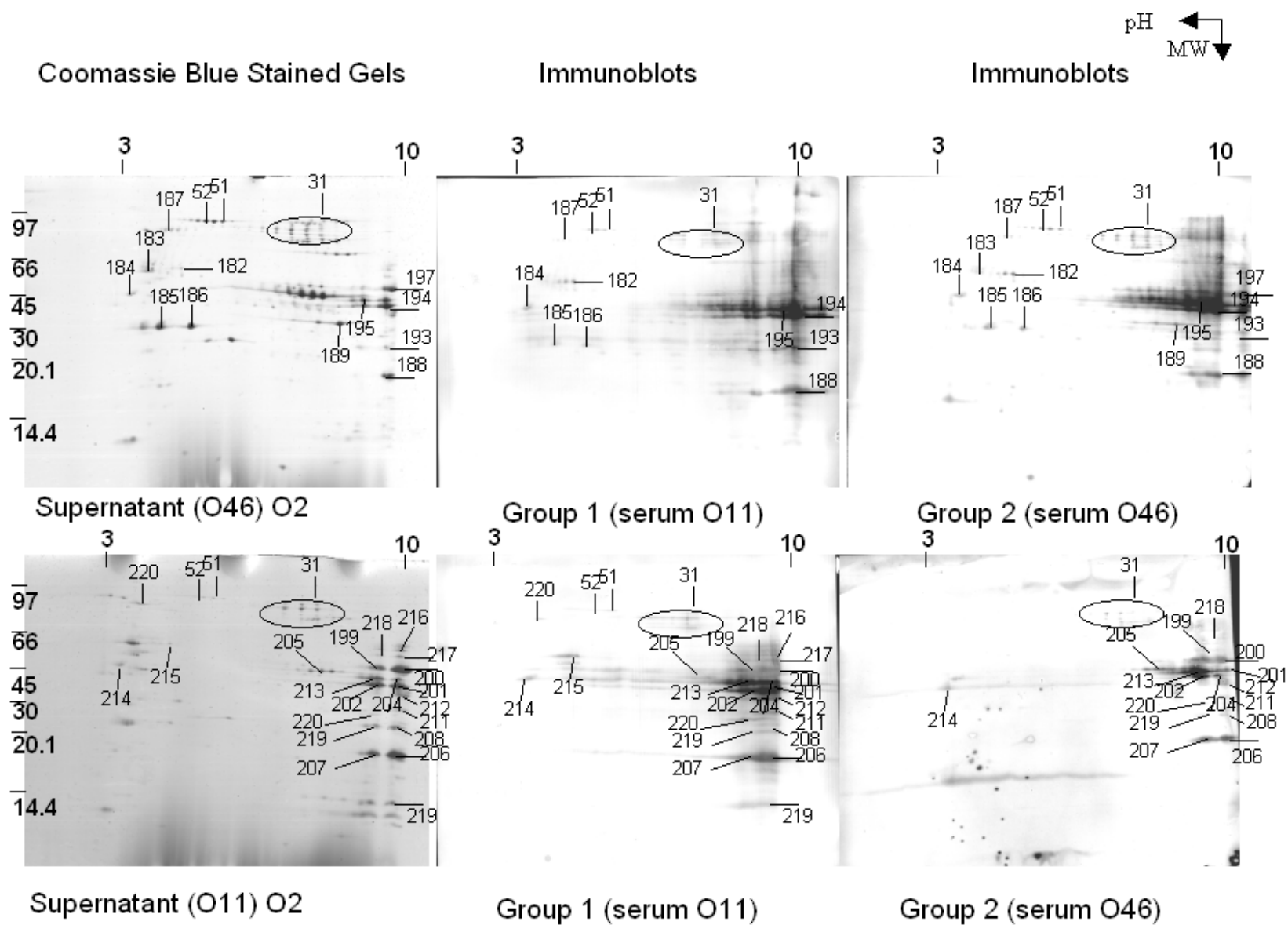

**Figure S6**

Supplement: Additional file 7 — Figure S6: Representative 2-DE gels and SERPA on supernatant fractions of S. aureus O46 (upper panels) and S. aureus O11 (lower panels). Supernatant samples were prepared from late exponential phase cultures of S. aureus strains grown aerobically on iron-depleted RPMI. Preparative 2-DE gels were Coomassie blue stained (left panel). Gels run in parallel were immunoblotted using the pools of sera obtained from group 1 (infected with O11) animals (middle panels) or from group 2 (infected with O46) animals (right panels). Samples were run in parallel on 13 cm gels (pI 3-10; 12% SDS-PAGE). Spots identified by MS/MS are labeled. [file 1297-9716-42-35-S7.PDF]
