# Supplementary material for: Staphylococcus aureus seroproteomes discriminate ruminant isolates causing mild or severe mastitis
Source: Vet Res. 2011 Feb 15;42(1):35. doi: 10.1186/1297-9716-42-35 (PMC3052181; doi:10.1186/1297-9716-42-35)
Supplement: Additional file 6 — Figure S5: Representative 2-DE gels and SERPA on cell wall fraction (upper panels) and total proteins (lower panels) of S. aureus O11. Supernatant samples were prepared from late exponential phase cultures of S. aureus strains grown anaerobically on iron-depleted RPMI. Preparative 2-DE gels were Coomassie blue stained (left panel). Gels run in parallel were immunoblotted using the pools of sera obtained from group 1 (infected with O11) animals (middle panels) or from group 2 (infected with O46) animals (right panels). Samples were run in parallel on 13 cm gels (pI 4-7; 12% SDS-PAGE). Spots identified by MS/MS are labeled. [file 1297-9716-42-35-S6.PDF]

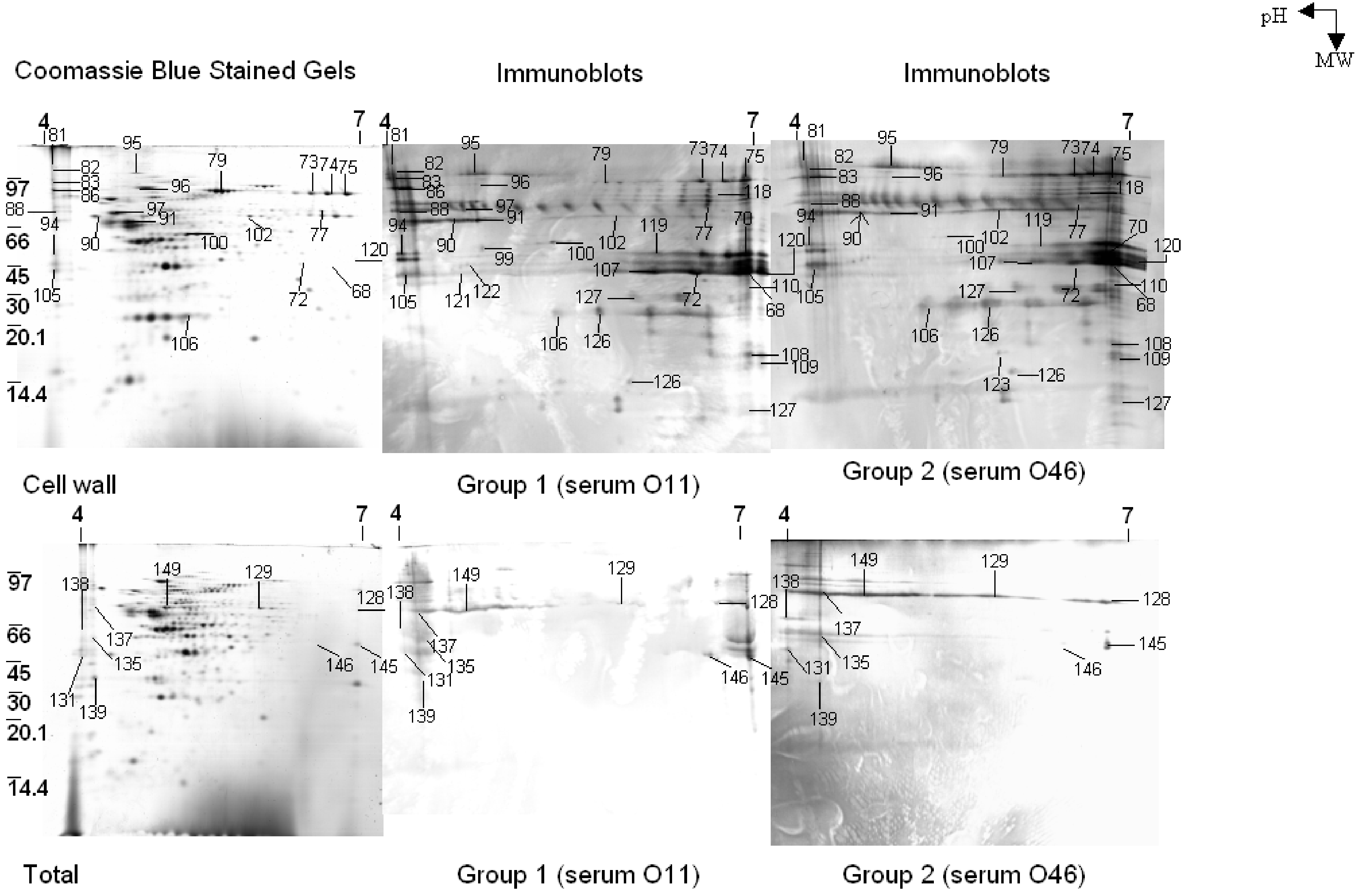

pH
MW
Coomassie Blue Stained Gels
Immunoblots
Immunoblots
97
66
45
30
20.1
14.4
Cell wall
Group 1 (serum O11)
Group 2 (serum O46)
Total
Group 1 (serum O11)
Group 2 (serum O46)

Figure S5
